# The Responsibility at Law of the Parent

**Published:** 1911-01-21

**Authors:** 


### The Hospital

A Journal of

The Medical Sciences and Hospital Administration.

New Series. No. 207, Vol. VIII [No. 1279, Vol. XLIX.] SATURDAY, JANUARY 21 1911.

### THE RESPONSIBILITY AT LAW OF THE PARENT.

In the annual report for the year 1909 of the Chief Medical Officer to the Board of Education there is so much that is of moment to the medical profession and to the political economist that it is difficult to decide which of the many topics discussed is the most instructive. The Report, too, is couched in language of such lucidity, moderation, and persuasiveness that it seems almost ungracious to take exception to any part of it; and, therefore, it may be worth stating at the outset that the greater part of it embodies facts and principles which most doctors can, and do, emphatically believe and support. It were to be desired that every citizen, whether a medical man or not, had time and opportunity to study this Blue-book; but since that is out of the question, no apology is necessary for adverting to Appendices B and D, in which the legal responsibility of the parent for attending to the health of his child is analysed.

It has been noted already in these pages that in fastening this responsibility on the parent, the most effective legislative weapon is provided by a Bill not intended to form part of the educational system of the country—namely, the Children Act. Under its provisions power is given to local authorities (including, of course, education authorities) to prosecute for neglect all parents who refuse to take measures to relieve their children of such preventable misery as is entailed by vermin, hunger, dirt, ear disease, lack of spectacles, etc. Several instances are quoted in Appendix B of prosecutions thus undertaken, and of convictions secured. Although in these cases repeated warnings had failed to relieve the gross neglect from which the children suffered, and penalties (usually very small) were inflicted, there is yet ground for believing that in an enormous number of cases the warnings were taken notice of, and that, temporarily at least, the condition of the unfortunate children of drunken or wastrel parents was improved.

Of greater importance to the medical profession is Appendix D, which deals with the recovery from parents of the cost, or part of the cost, of treatment ordered by school medical officers. Under the Medical Treatment Act of 1909 it is obligatory on the part of a local education authority to charge the parent a sum not exceeding that actually expended unless by reason of circumstances other than his own default he is unable to pay the amount. It is easily understood that when hundreds of children are under treatment, whether it be at a subsidised hospital, in a school clinic, or by any other scheme financed from public funds, to determine the cost per caput per attendance is, to begin with, difficult. Not only is it difficult, it is also of no avail. For eminent legal authorities have given their opinion that to average out the cost in this way is not permissible for the purpose of charging the parent. The sum demanded of the latter must represent, as nearly as possible, the actual cost to the authority of the particular child in question. It is undesirable, says the Report, to differentiate in the charges for treatment at different hospitals, but it is possible to vary the charges made in respect of various ailments. Thus it has been calculated that the average cost of treating at a subsidised hospital each case of eye disease is four shillings, while that of ear disease is five shillings. The average number of attendances per patient is about three; hence the cost per attendance is in the former cases one shilling and fourpence, and in the latter class one shilling and eightpence.

How much of this expense can be, and is likely to be, recovered from parents there are as yet no reliable data to judge from. Where the parents can afford to pay the full amount they should be required to do so; and in other cases a proportion should be demanded, and enforced if necessary, according to the circumstances of each particular case. The London County Council has taken this matter into detailed consideration, and has devised a scale for determining the payments to be made according to the income of the family and the number of individuals who compose it. The standard family is regarded as consisting of two adults and four children of school age. Each child under fourteen is reckoned as equivalent to 0.75 of an adult; so the standard family is thus taken as five adults. When there are more in the family than this the wages earned are divided, for the purposes of classification, by the number of adults in the family, and multiplied by five. Thus in a family larger than the standard the actual wage is reduced, and so the payment to be made is calculated on a lower scale. In addition to this it is to be noticed that the income of the family is the net income; that is to say, rent, insurance, sick-club subscriptions, and fares are subtracted from it. When the income is classified as below seventeen shillings and sixpence a week no charge is made; between that sum and twenty shillings a week fourpence per attendance is the sum collected; between a sovereign and twenty-two and six, eightpence; between twenty-two and six and twenty-five shillings, one shilling; under twenty-seven and six, one and fourpence; over twenty-seven and six, one and fourpence for eye cases, one and eightpence for ear cases. In no case are more than three attendances to be charged for. This last proviso seems to be a great mistake. Take the case of a skilled mechanic earning three or four or five pounds a week, as many of them do without difficulty, especially in the north of England; let us suppose he has two children who attend a Board school. That man can well afford to pay one and eightpence a week for many weeks if his child has an obstinate otorrhœa; and to relieve him of all responsibility in the matter for the sum of five shillings is fair neither to the ratepayer, to the hospital, nor to the profession. But in the main the scale seems to hit fairly well the happy mean between pauperisation, with destruction of parental responsibility, on the one hand, and any suspicion of a grinding of the faces of the poor upon the other. Once adopted, it should be unfalteringly adhered to.

